# Absolute Risks and Decision Tools for Communicating the Risks of Visual Impairment From Myopia-Related Diseases

**DOI:** 10.1167/iovs.66.4.82

**Published:** 2025-04-30

**Authors:** Emma Dow, Stephanie Kearney, Mhairi Day

**Affiliations:** 1 Department of Vision Sciences, School of Health and Life Sciences, Glasgow Caledonian University, Glasgow, Scotland, United Kingdom

**Keywords:** myopia, visual impairment, complications, risk

## Abstract

**Purpose:**

The risks of developing myopia complications are frequently reported in relative terms, which can be misleading. This study provides absolute risk estimates of visual impairment (VI) from myopia-related diseases.

**Methods:**

A critical integrative review provided data on frequency of myopic macular degeneration (MMD), primary open-angle glaucoma (POAG), rhegmatogenous retinal detachment (RRD), and associated VI in predominantly White and East Asian populations. The absolute risks of persons over 40 years of age with no myopia, low myopia (−2.00 D), or high myopia (−6.00 D) developing VI from each myopia-related disease were calculated by multiplying the proportion of each refractive group with the disease by the rate of VI. The sum of the risks of VI from MMD, POAG, and RRD provided an estimate of VI risk from any of these three myopia-related diseases in adults over 40 years old.

**Results:**

VI from MMD, POAG, or RRD combined is expected in 0.4 in 100, 1.4 in 100, and 6.8 in 100 of White persons with no myopia, low myopia, or high myopia, respectively. The same risks in an East Asian population are 0.5 in 100, 2.4 in 100 and 10.3 in 100 in persons with no myopia, low myopia, or high myopia, respectively.

**Conclusions:**

Absolute risks are provided to enable balanced discussions of the future risk that a child may have in developing VI from myopia-related diseases when considering myopia management. These estimates should be put into context using decision tools and balanced statements providing information on the likelihood of both developing VI and not developing VI.

The risks of myopia to ocular health have been well documented, with several studies highlighting the association between myopia and sight-threatening ocular pathologies such as rhegmatogenous retinal detachment (RRD),^1^^–^^3^ primary open-angle glaucoma (POAG),^4^^,^^5^ and myopic macular degeneration (MMD).^6^^,^^7^ All individuals with myopia are at risk of developing these diseases; however, the risk is greatest for those with high levels of myopia (spherical equivalent refraction [SER] ≤ −6.00) due to the longer axial length of the eye.^8^ The development of myopia-related ocular pathologies significantly affects quality of life^9^^,^^10^ and has a number of financial implications, such as the direct costs of managing and treating the disease and the indirect costs of time spent receiving care.^11^^,^^12^ However, arguably, it is the potential visual impairment (VI) from myopia that has the greatest economic impact. In addition to the costs associated with managing MMD, POAG, and RRD, the development of VI from these conditions leads to further spending on the provision of low-vision rehabilitation, community care, and potential loss of workplace productivity.^12^^–^^14^ The prevention of VI and blindness caused by myopia is outlined within the mission statement of the International Myopia Institute (IMI)^15^ and has driven research into myopia management strategies. Myopia management refers to specialized treatments to slow the progression of myopia, such as dual-focus contact lenses,^16^ specialized spectacle lenses,^17^^,^^18^ orthokeratology,^19^ and pharmacological agents.^20^ These interventions have demonstrated success in reducing the rate of axial growth in children compared to standard single-vision correction,^16^^–^^20^ and are prescribed by a number of eye health professionals for the management of childhood myopia.^21^ Slowing the rate of axial length progression may reduce the likelihood of a child developing high myopia, and in turn, reduce their risk of developing myopia-related ocular pathologies and subsequent VI.

The likelihood of a person with myopia developing a myopia-related ocular pathology can be described in relative or absolute terms. Relative risk is a measure of how much more (or less) risk an individual in one group has of an event occurring compared to an individual in another group.^22^ In this context, relative values demonstrate how much more risk an individual with myopia has of developing VI from a myopia-related disease compared to an individual without myopia. However, relative values do not provide information on what the likelihood of developing the complication is in each group (i.e., the absolute risk).^22^ Absolute risk refers to the proportion of people that experience an event within a specific population.^22^ For example, the absolute risk of a myopic person developing VI from a myopia-related disease is the proportion of people with myopia who develop VI from a myopia-related disease.

In the literature, the probability of an individual developing a myopia-related complication is often described in relative terms.^1^^,^^5^^,^^8^^,^^23^^–^^25^ Relative measures are important for assessing the strength of an association between a risk factor and disease; however, data on the absolute likelihood of developing the disease are necessary to provide context and appropriately interpret the clinical significance of a risk factor.^26^ For example, a recent meta-analysis reported that the odds ratios (ORs) of persons with low myopia (SER ≤ −0.50 to > −3.00) and those with moderate or high myopia (SER ≤ −3.00) developing POAG are 1.59 and 2.92, respectively.^8^ This information tells us that the odds of an individual with high myopia developing POAG are almost three times greater than for a non-myopic person; however, without data on the frequency of POAG in the non-myopic population, it is not possible to fully interpret the absolute probability of a person with high myopia developing POAG. Another paper reporting relative measures of risk stated that the prevalence of MMD increases by 67% for each 1-diopter (D) increase in myopia,^24^ indicating that an individual with −3.00 D of myopia will have a 67% greater risk of developing MMD compared to a person with −2.00 D of myopia. However, if the baseline risk of MMD is low, an increase of 67% will not drastically alter the absolute risk. Indeed, the reported prevalence of MMD in people with low myopia is between 0.7% and 7%.^7^^,^^27^^,^^28^ Increasing these values by 67% results in a prevalence of between 1.2% and 11.7%, which represent absolute risk differences of 0.5% and 4.7%, respectively.

Solely reporting relative probabilities can be misleading, as doing so may exaggerate the perception of disease risk.^29^ Research initiatives and healthcare bodies such as the Strengthening the Reporting of Observational Studies in Epidemiology (STROBE) statement and the UK National Institute for Health and Care Excellence (NICE) advocate the use of absolute values over ratios.^26^^,^^30^ In addition, guidance from the UK College of Optometrists affirms that the potential reduction in disease risk that can be achieved with myopia management “… needs to be considered in context with respect to absolute risk, as the incidence of these complications in the general population is low.”^31^ Despite this, many publications emphasize the relative risk of developing myopia-related diseases while providing little information on absolute risk. Furthermore, not all individuals with myopia-related diseases will develop VI from their condition, and the prevalence of VI from myopia complications is an important consideration when assessing the potential impact of myopia on an individual and societal level. Although there are studies reporting the prevalence of MMD, POAG, and RRD and the prevalence of VI from these diseases, the combined risk of developing these diseases and subsequent VI has not been reported as an absolute value. Therefore, this paper aims to provide estimates of the absolute risks of MMD, POAG, and RRD and subsequent VI in different population groups to illustrate the importance of using absolute over relative values when communicating the risk of myopia-related complications in practice. These risk estimates can be used by clinicians to enable balanced discussions with parents or caregivers and their children when considering the future risk that the child may have in developing VI as a result of an eye disease due to myopia, as well as the potential benefits of myopia management interventions.

## Methods

### Literature Review

A critical integrative review was conducted to gather data on the risk of an individual developing MMD, RRD, POAG, and associated VI. These conditions were chosen as they are the most prevalent myopia-related complications.^8^ The literature review was performed on the ProQuest MEDLINE database using a combination of search terms such as “myopia complications,” “visual impairment,” “visual outcomes,” “retinal detachment,” “rhegmatogenous,” “myopic macular degeneration,” “myopic maculopathy,” “glaucoma,” and “primary open angle.” Only peer-reviewed research papers were included. Where possible, data were obtained from observational, epidemiological studies. Data on the geographical region and time period of data collection, participant demographics, disease definition, VI definition, and prevalence of disease and/or VI were collected from each study. Studies reporting the disease prevalence within adult populations 40 years of age and over were selected to ensure that the risk estimates represented the time period at which myopia-related complications are likely to present. Studies with a small sample size were excluded to ensure that data were representative of a more generalized population.

In order to report the disease risk for a range of patient groups while also summarizing the findings in a concise and manageable way, the risk data were selected for three refractive levels: no myopia, a representative value of low myopia (SER of −2.00 D), and a representative value of high myopia (SER of −6.00 D). The data were reported for two population groups: White and East Asian. Studies conducted in Europe and Australia were categorized as predominantly White populations and those conducted in China and Singapore were categorized as East Asian populations. There were insufficient data identified in the literature review to report risk estimates for other population groups. The definition of “no myopia” varied among the studies included in the final analysis, but it was most commonly defined as a SER > −1.00 D. Unless otherwise stated, this definition was used to categorize non-myopic cases. The definition of VI varied slightly among different studies, but most adhered to the International Classification of Diseases 11th Revision (ICD-11) threshold for mild VI, which is defined as a visual acuity (VA) worse than 0.3 logMAR.^32^

Fourteen papers reporting the prevalence of MMD were identified in the literature review.^7^^,^^27^^,^^28^^,^^33^^–^^43^ Nine papers were excluded due to absent or insufficient information on myopia severity,^33^^–^^36^^,^^38^^,^^39^^,^^43^ disease prevalence,^41^ or VI prevalence.^40^ Two papers were excluded as the participants did not meet the correct age criteria.^37^^,^^42^ In the final analysis, three population-based research studies were included which were conducted in Australia (Blue Mountains Eye Study^7^), Singapore (Singapore Epidemiology of Eye Diseases^27^), and Beijing (Beijing Eye Study^28^). The details of these studies are summarized in Table 1. All three studies provided data on the prevalence of MMD categorized by refractive error severity, as well as the frequency of VI among subjects with MMD.

**Table 1.** Studies Included in the Analysis of MMD Risk

Study	Study Population	MMD Classification^*^	MMD Prevalence by Refractive Category (SER)	VI Due to MMD
Blue Mountains Eye Study^7^	*N* = 3583White, AustraliaEthnicity, 99% White^67^Age: 49+ y	Myopic retinopathy was classified by presence of staphyloma, lacquer cracks, Fuchs spot, myopic chorioretinal thinning, or atrophy	Overall: 1.2% (*n* = 44)>−1.00 D: 0.3%−1.00 to −2.99 D: 0.7%−3.00 to −4.99 D: 3%−5.00 to −6.99 D: 11.4%−7.00 to −8.99 D: 28.6%<−9.00 D: 52.4%	38.8% of affected eyes with VA > 0.3 logMAR
Beijing Eye Study^28^	*N* = 4439East Asian, ChinaAge: 40+ y	Myopic retinopathy was defined by the presence of staphyloma, lacquer cracks, Fuchs spot, or chorioretinal atrophy	Overall: 3.1% (*n* = 132; 198 eyes)−0.50 to −1.99 D: 0%−2.00 to −3.99 D: 3.8%−4.00 to −5.99 D: 15.9%−6.00 to −7.99 D: 40.4%−8.00 to −9.00 D: 72.9%≤−10.00 D: 89.6%	16.6% of affected participants with VA > 0.3 logMAR to ≤1.0 logMAR and 1.5% with VA >1.0 logMAR
Singapore Epidemiology of Eye Disease Study^27^	*N* = 8716East Asian, SingaporeEthnicity: 33.6% Malay, 33.1% Indian, and 33.3% ChineseAge: 40+ y	META-PM classification was used.^68^ Eye was considered to have MMD if grade 2, 3, or 4 or presence of any “plus” lesions	Overall: 3.8% (n = 350)≤−0.50 to >−3.00 D: 7.0%≤−3.00 and >−5.00 D: 10.4%≤−5.00 and >−8.00 D: 17.1%≤−8.00 D: 53.3%	21.8% of participants with MMD had VA >0.3 logMAR in at least one eye

* Note that some studies used the term “myopic retinopathy” in lieu of MMD.

Ten papers were identified in the literature review that reported the prevalence of glaucoma within different refractive groups.^4^^,^^44^^–^^52^ As the aim of this paper is to report the risk of POAG, studies that did not differentiate among the different types of glaucoma (e.g., secondary, angle-closure glaucoma) were excluded.^44^^,^^46^ Five papers were excluded due to limited information on prevalence and/or myopia severity.^45^^,^^48^^,^^50^^–^^52^ Subsequently, the three studies that were included in the analysis of POAG risk were conducted in Australia (the Blue Mountains Eye Study^4^) and Singapore (Singapore Malay Eye Study^47^ and Singapore Indian Eye Study^49^). A summary of each study included in the analysis of POAG risk is provided in Table 2. The prevalence of VI and blindness in subjects with glaucoma in the Singapore Malay Eye Study was reported in a separate paper^53^; the percentage of eyes with POAG and VI outlined in this study was used to determine the risk of VI due to POAG in Singaporean individuals. The prevalence of VI from POAG was not reported in the Blue Mountains Eye Study,^4^ and there were no alternative research papers identified in the literature search that reported the prevalence of VI in Australian subjects with glaucoma. Therefore, data on VI in a predominantly White population were obtained from a study conducted in the United Kingdom that assessed the lifetime risk of VI due to visual field loss in glaucoma patients.^54^

**Table 2.** Summary of Studies Used to Assess the Risk of POAG

Study	Study Population	POAG Classification	POAG Prevalence by Refractive Category (SER)	VI Due to POAG
Blue Mountains Eye Study^4^	*N* = 3654White, AustraliaEthnicity: 99% White^67^Age: 49+ y	Presence of optic disc cupping with rim thinning (cup-to-disc ratio ≥ 0.7 or cup-to-disc asymmetry ≥ 0.3) and characteristic visual field loss on automated perimetry in the absence of angle closure or secondary causes of glaucoma	>−1.00 D: 1.5%≤−1.00 to >−3.00 D: 4.2%≤ −3.00 D: 4.4%	Not reported
Singapore Malay Eye Study^47^	*N* = 3280East Asian, SingaporeEthnicity: MalayAge: 40–80 y	POAG was classified using the International Society for Geographical and Epidemiological Ophthalmology categories 1, 2, and 3,^69^ in the presence of an open anterior chamber angle	>+0.50 D: 2%+0.50 to −0.50 D: 1.4%<−0.50 to −4.00 D: 2.6%<−4.00 D: 4%	Not reported
Singapore Indian Eye Study^49^	*N* = 3400East Asian, SingaporeEthnicity: IndianAge: 40−84 y	POAG was classified using the International Society for Geographical and Epidemiological Ophthalmology categories 1, 2, and 3,^69^ in the presence of an open anterior chamber angle (pigmented trabecular meshwork observed for 270° or more of the angle circumference during gonioscopy) and absence of secondary pathologies	>+0.50 D: 0.8%≤+0.50 to ≥−0.50 D: 1%<−0.50 to ≥−3.00 D: 0.7%<−3.00 to ≥−6.00 D: 1%<−6.00 D: 5.3%	Not reported

There were no epidemiological studies reporting the frequency of RRD in a myopic population identified in the literature review. Seven studies reporting the frequency of RRD and/or the relationship between refractive error and RRD were reviewed.^1^^,^^2^^,^^55^^–^^59^ Two studies were excluded from the analysis of RRD risk due to absent or insufficient refractive data,^56^^,^^57^ and three studies were considered to have limited generalizability as a result of the study design or sample.^1^^,^^55^^,^^58^ The remaining two papers were population-based epidemiological studies reporting the incidence of RRD in Beijing^59^ and Scotland.^2^ Both studies included data on the refractive distribution of RRD cases. A summary of the studies included in the analysis of RRD risk can be found in Table 3.

**Table 3.** Summary of Studies Used to Assess the Risk of RRD

Study	Population	RRD Classification	Annual RRD Incidence	SER Breakdown of RRD Cases	VI Due to RRD
Scottish Retinal Detachment Study^2^	White, Scotland, UKEthnicity: 98% White British*N* = 5,168,5001244 RRD cases over 2-y periodAll ages	Area of subretinal fluid greater than two disc diameters in size with a full thickness retinal break	12.05 per 100,000	≥+6.00 D: 0.8%>+1.00 to <+6.00 D: 8.6%≥−1.00 to ≤+1.00 D: 29.2%<−1.00 to >−6.00 D: 35.1%≤−6.00 D: 18.1%Unknown: 8.2%	Not reported
Beijing RRD Study Group^59^	East Asian, Beijing, China*N* = 6,589,000519 RRD cases over 1 yAll ages	Retinal elevation with any retinal break and subretinal fluid extended one or more disc diameters from the break margin	7.98 per 100,000	Non-myopic: 33.5%−1.00 to > −6.00 D:29.7%≤ −6.00 D: 33.8%	Not reported

To estimate the risk of RRD in an individual with myopia, data on the prevalence of myopia within the general population, population size, and life expectancy were required. In the Scottish Retinal Detachment Survey, Mitry et al.^2^ referred to the 2008 mid-year population estimates from the National Records of Scotland (formerly known as the General Register Office for Scotland) to calculate annual RRD incidence. The 2008 mid-year population estimates in Scotland have since been updated and were consulted to provide data on population size grouped by age.^60^ Age-stratified population data from Beijing were extrapolated from the Beijing Rhegmatogenous Retinal Detachment Study Group paper.^59^ The prevalence of myopia among Scottish adults was estimated using data from the UK Biobank,^61^ and myopia prevalence in Beijing was projected using data from the Liwan Eye Study.^62^ The life expectancies of the population in Scotland and Beijing were obtained from the National Records of Scotland^63^ and Yang et al.,^64^ respectively. The literature review did not reveal any studies that documented the frequency of VI from RRD in China. Therefore, data on VI risk from RRD in East Asian individuals were obtained from a paper that reported the postsurgical visual outcomes of RRD patients in Singapore, of which almost 90% were of Chinese ethnicity.^65^ Data on the risk of VI from RRD in Scottish people were obtained from two studies that reported the frequency of macula-off retinal detachments and visual outcomes of macula-off retinal detachments in Scotland and the wider UK population, respectively.^56^^,^^66^

### Risk Calculation

The risk of developing VI from each myopia-related disease was calculated using the formula provided in Equation 1:

(1) $$\begin{array}{ccc} & & \;\mathrm{Risk}\;\mathrm{of}\;\mathrm{disease}\;and\;\mathrm{VI}\\ & & \;=\mathrm{Proportion}\;\mathrm{of}\;\mathrm{refractive}\;\mathrm{group}\;\mathrm{with}\;\mathrm{the}\;\mathrm{disease}\\ & & \;\;\times \;\mathrm{Prevalence}\;\mathrm{of}\;\mathrm{VI}\;\mathrm{from}\;\mathrm{the}\;\mathrm{disease} \end{array}$$

For MMD and POAG, the proportion of myopic persons with the disease and prevalence of VI from the disease were extracted directly from the studies identified within the literature review. Each study provided the prevalence of the disease within a defined refractive category (e.g., −1.00 to −2.99 D). The disease prevalence for low and high myopia was determined by selecting data from the refractive category that encompassed a SER of −2 D and −6 D, respectively. The selection of the disease prevalence for the “no myopia” category was based on the definition of “no myopia” by individual papers; as previously stated, for most studies it was defined as a SER > −1.00 D. Risk estimates that were calculated based on an alternative definition of “no myopia” have been highlighted within the relevant tables in the Results section. The risk of VI from MMD in non-myopic persons was not calculated, as MMD only occurs in individuals with myopia.

Due to the lack of epidemiological studies on the frequency of retinal detachment in a myopic population, a number of additional steps were required to calculate the proportion of people in each refractive category with RRD (Equations 2 and 3). The papers identified in the literature review^2^^,^^59^ reported the annual incidence and refractive distribution of RRD cases. To determine the number of RRD cases that occur in myopic and non-myopic individuals across an average lifetime, the annual incidence of RRD was multiplied by the proportion of RRD cases with the relevant refractive error, population size, and remaining life expectancy, as noted in Equation 3. As the incidence of RRD is uncommon before the age of 40 years,^2^ only cases that occurred from the age of 40 years onward were counted to represent the risk of developing RRD. The remaining number of years an individual would be at risk of RRD was determined by subtracting 40 years from the life expectancy of the population. The total number of people with no myopia, low myopia, or high myopia in the population was estimated by multiplying the population size by the respective prevalence of no myopia (emmetropia and hyperopia), low myopia, and high myopia in the population:

(2) $$\begin{array}{ccc} & & \;\mathrm{Proportion}\;\mathrm{of}\;\mathrm{refractive}\;\mathrm{group}\;\mathrm{with}\;\mathrm{RRD}\\ & & \;=\frac{\begin{array}{c} \mathrm{Number}\;\mathrm{of}\;\mathrm{RRD}\;cases\;\mathrm{in}\;\mathrm{each}\;\mathrm{refractive}\;\mathrm{group} \end{array}}{\begin{array}{c} \mathrm{Total}\;\mathrm{number}\;\mathrm{of}\;\mathrm{people}\;\mathrm{in}\;\mathrm{the}\;\mathrm{refractive}\;\mathrm{group} \end{array}} \end{array}$$

(3) $$\begin{array}{ccc} & & \;\mathrm{Number}\;\mathrm{of}\;\mathrm{RRD}\;\mathrm{cases}\;\mathrm{in}\;\mathrm{each}\;\mathrm{refractive}\;\mathrm{group}\\ & & \;=\mathrm{Annual}\;\mathrm{incidence}\;\mathrm{of}\;\mathrm{RRD}\;\;\times \;\mathrm{Proportion}\;\mathrm{of}\;\mathrm{RRD}\\ & & \;\;\mathrm{cases}\;\mathrm{with}\;\mathrm{each}\;\mathrm{refractive}\;\mathrm{error}\;\times \;\mathrm{Population}\;\mathrm{size}\\ & & \;\;\times \;\mathrm{Remaining}\;\mathrm{life}\;\mathrm{expectancy} \end{array}$$

To calculate the combined risk of RRD and subsequent VI, the risk of developing RRD was multiplied by the prevalence of VI following surgical treatment for RRD (Equation 1). The proportion of RRD cases that result in VI in an East Asian population was obtained directly from a study by Rosman et al.^65^ VI data for predominantly White subjects was inferred from two RRD studies conducted in the United Kingdom.^56^^,^^66^ The most recent Scottish Retinal Detachment Survey^56^ provided data on the proportion of RRD cases that involve macular detachment, which the authors noted had remained stable since the last Scottish Retinal Detachment Survey,^2^ from which RRD incidence data were obtained for the risk calculation. An earlier study by Yorston et al.^66^ reported the number of eyes with macula-off retinal detachments that achieved a VA of 0.3 logMAR or better post-surgery. Assuming that only macula-off retinal detachments result in poor central VA, the risk of VI from RRD was calculated by multiplying the proportion of all RRD cases that are considered “macula-off” by the proportion of macula-off RRD cases with a postoperative VA worse than 0.3 logMAR.

The risks of disease and subsequent VI for MMD, POAG, and RRD were added together to obtain the probability of developing VI from any of these three myopia-related pathologies (Equation 4):

(4) $$\begin{array}{ccc} & & \;\mathrm{Risk}\;\;\mathrm{of}\;\;\mathrm{VI}\;\;\mathrm{from}\;\mathrm{MMD},\;\mathrm{POAG},\;\mathrm{or}\;\;\mathrm{RRD}\\ & & \;=\;\mathrm{Risk}\;\;\mathrm{of}\;\;\mathrm{MMD}\;\;\mathrm{and}\;\mathrm{VI}+\;\mathrm{Risk}\;\;\mathrm{of}\;\;\mathrm{POAG}\;\mathrm{and}\;\mathrm{VI}\\ & & \;\;+\;\mathrm{Risk}\;\;\mathrm{of}\;\;\mathrm{RRD}\;\;\mathrm{and}\;\mathrm{VI} \end{array}$$

As there were two separate estimates provided for “risk of MMD and VI” and “risk of POAG and VI” in East Asian populations, the highest risk estimates for each disease were selected in the calculation of the risk of VI from MMD, POAG, or RRD in an East Asian population. Because non-myopic persons are not susceptible to MMD, a value of zero was assigned to “risk of MMD and VI” for this refractive group. In alignment with NICE guidelines, all absolute risk estimates have been presented in natural frequencies (e.g., 1 in 100).^30^ As the data from the epidemiological studies used for these calculations were obtained from adult populations over 40 years old, the figures provided represent the risks of VI from myopia-related diseases for adults over 40 years old. This broad time frame encompasses the entire period during which a person is likely to develop a myopia-related disease and, therefore, is appropriate in the context of facilitating discussions between clinicians and parents or caregivers about a child's future risk of VI due to myopia.

## Results

### Myopic Macular Degeneration

A summary of the results is provided in Table 4. The risks of MMD for persons with low and high myopia from Australia are 0.7 in 100 (0.7%) and 11.4 in 100 (11.4%), respectively.^7^ It was reported that 38.8% of eyes affected by MMD in the Australian Blue Mountains Eye Study were classified as visually impaired (VA > 0.3 logMAR).^7^ As a result, the overall risk of VI due to MMD in Australia is 0.3 in 100 for a person with low myopia and 4.4 in 100 for a person with high myopia. Similarly, the risk of MMD and the risk of MMD and VI for a person with low myopia living in China^28^ is 3.8 in 100 and 0.7 in 100, respectively. A person with high myopia in China^28^ has a 40.4 in 100 risk of developing MMD and a 7.3 in 100 risk of MMD and VI. Absolute risk data for Singapore^27^ are also provided in Table 4.

**Table 4.** Absolute Risks of Developing MMD and VI Due to MMD in Australia, China, and Singapore

Population	Refractive Group	Proportion of People With Myopia Who Develop MMD	Proportion of MMD Cases Resulting in VI	Proportion of People With MMD and VI
White, Australia	Low myopia	0.7 in 100	38.8 in 100	0.3 in 100
	High myopia	11.4 in 100		4.4 in 100
East Asian, Beijing, China	Low myopia	3.8 in 100	18.1 in 100	0.7 in 100
	High myopia	40.4 in 100		7.3 in 100
East Asian, Singapore	Low myopia	7.0 in 100	21.8 in 100	1.5 in 100
	High myopia	17.1 in 100		3.7 in 100

### Primary Open-Angle Glaucoma

In Australian subjects, the risks of POAG in persons with no myopia, low myopia, or high myopia were 1.5 in 100 (1.5%), 4.2 in 100 (4.2%), and 4.4 in 100 (4.4%), respectively.^4^ In Singapore Malay subjects, 1.7 in 100 non-myopic persons, 2.6 in 100 persons with low myopia, and 4.0 in 100 persons with high myopia are at risk of POAG.^47^ The risks of POAG in Singapore Indian subjects is 0.9 in 100, 0.7 in 100, and 1.0 in 100 persons with no myopia, low myopia, or high myopia, respectively.^49^

According to Shen et al.,^53^ 16.3% of eyes with POAG in the Singapore Malay Eye Study were classified as visually impaired (VA, >0.30 to <1.00 logMAR), and 5.8% were classified as blind (VA ≥ 1.00 logMAR). In total, 22.1% of eyes had a VA > 0.3 logMAR. Subsequently, the risks of POAG and VI (VA > 0.3 logMAR) among Singapore Malay individuals with no-myopia, low myopia, or high myopia are 0.4 in 100, 0.6 in 100, and 0.9 in 100, respectively. Using VI data from the Singapore Malay Eye Study,^53^ it was calculated that the risk of POAG and VI in Singapore Indian subjects is 0.2 in 100 for persons with no myopia, low myopia, or high myopia. Visual field series data from a UK-based study was used to estimate the risk of VI from POAG in a predominantly White population.^54^ Saunders et al.^54^ used visual field series from 3790 patients attending glaucoma clinics in UK hospitals to calculate the rate of visual field loss and risk of VI across the patient's remaining lifetime. VI was classified as a mean deviation (MD) of −14 dB or worse, and the authors reported a best-case scenario and a worst-case scenario for VI risk based on a patient with either slow or fast progression of visual field loss. The proportion of glaucoma patients expected to develop VI in their lifetime is 10.0% in a best-case scenario and 11.5% in a worst-case scenario.^54^ Using the worst-case scenario value, the overall risks of VI from POAG in persons with no myopia, low myopia, or high myopia from a predominantly White population are 0.2 in 100, 0.5 in 100, and 0.5 in 100, respectively. A summary of the risk of POAG and the risk of VI from POAG is provided in Table 5.

**Table 5.** Absolute Risks of Developing POAG and VI Due to POAG in Australia and Singapore

Population	Refractive Group	Proportion of People Who Develop POAG	Proportion of POAG Cases Resulting in VI	Proportion of People With POAG and VI
White, Australia	No myopia	1.5 in 100	11.5 in 100^*^	0.2 in 100
	Low myopia	4.2 in 100		0.5 in 100
	High myopia	4.4 in 100		0.5 in 100
East Asian, Singapore (Malay)	No myopia^†^	1.7 in 100	22.1 in 100	0.4 in 100
	Low myopia	2.6 in 100		0.6 in 100
	High myopia	4.0 in 100		0.9 in 100
East Asian, Singapore (Indian)	No myopia^†^	0.9 in 100	22.1 in 100^‡^	0.2 in 100
	Low myopia	0.7 in 100		0.2 in 100
	High myopia	1.0 in 100		0.2 in 100

* Data from a UK-based study.^54^

† “No myopia” was defined as SER ≥ −0.50 D.

‡ Data from Singapore Malay Eye Study.^53^

### Rhegmatogenous Retinal Detachment

The annual incidences of RRD cases in Scotland that occur in people with no myopia, low myopia, or high myopia over the age of 40 years are 19 per 100,000, 55 per 100,000, and 161 per 100,000, respectively. Across the average lifetime, the risks of RRD in Scotland are 0.7 in 100 for a non-myopic person, 2.1 in 100 for a person with low myopia, and 6.2 in 100 for a person with high myopia. In the most recent Scottish Retinal Detachment Survey, it was reported that 58% of RRD cases involve detachment of the macula.^56^ Yorston et al.^66^ found that 48.8% eyes with a macula-off RRD achieve a postoperative VA of 0.3 logMAR or better, suggesting that 51.2% of eyes have a postoperative VA worse than 0.3 logMAR. Using the criteria of VA worse than 0.3 logMAR as the threshold for VI, and assuming that only macula-off retinal detachments result in poor central VA, it was determined that 29.7% of RRD cases develop VI. Consequently, the combined risk of developing RRD and VI for a person with no myopia, low myopia, or high myopia living in Scotland is 0.2 in 100, 0.6 in 100, and 1.9 in 100, respectively.

In China, the annual incidences of RRD in persons with no myopia, low myopia, or high myopia over 40 years old in Beijing are 6 per 100,000, 14 per 100,000, and 84 per 100,000, respectively. It is estimated that across the average lifetime 0.3 in 100 persons with no myopia, 0.6 in 100 persons with low myopia, and 3.6 in 100 persons with high myopia in China develop RRD. A study of Singaporean RRD found that 59.7% of RRD cases had a postoperative VA of <6/18 (0.5 logMAR).^65^ Using this data as a representation of the risk of VI following RRD surgery in East Asia, it was determined that the respective proportions of persons with no myopia, low myopia, or high myopia in China that develop RRD and subsequent VI are 0.2 in 100, 0.3 in 100, and 2.1 in 100. A summary of these findings is provided in Table 6.

**Table 6.** Absolute Risks of Developing RRD and VI Due to RRD in Scotland and China

Population	Refractive Group	Proportion of People Who Develop RRD	Proportion of RRD Cases Resulting in VI	Proportion of People With RRD and VI
White, Scotland, UK	No myopia^*^	0.7 in 100	29.7 in 100	0.2 in 100
	Low myopia	2.1 in 100		0.6 in 100
	High myopia	6.2 in 100		1.9 in 100
East Asian, Beijing, China	No myopia	0.3 in 100	59.7 in 100^†^	0.2 in 100
	Low myopia	0.6 in 100		0.3 in 100
	High myopia	3.6 in 100		2.1 in 100

* “No myopia” was defined as SER ≥ −1.00 D.

† Data from a Singapore-based study.^65^

### Risk of VI from MMD, POAG, or RRD

**Figure 1.**
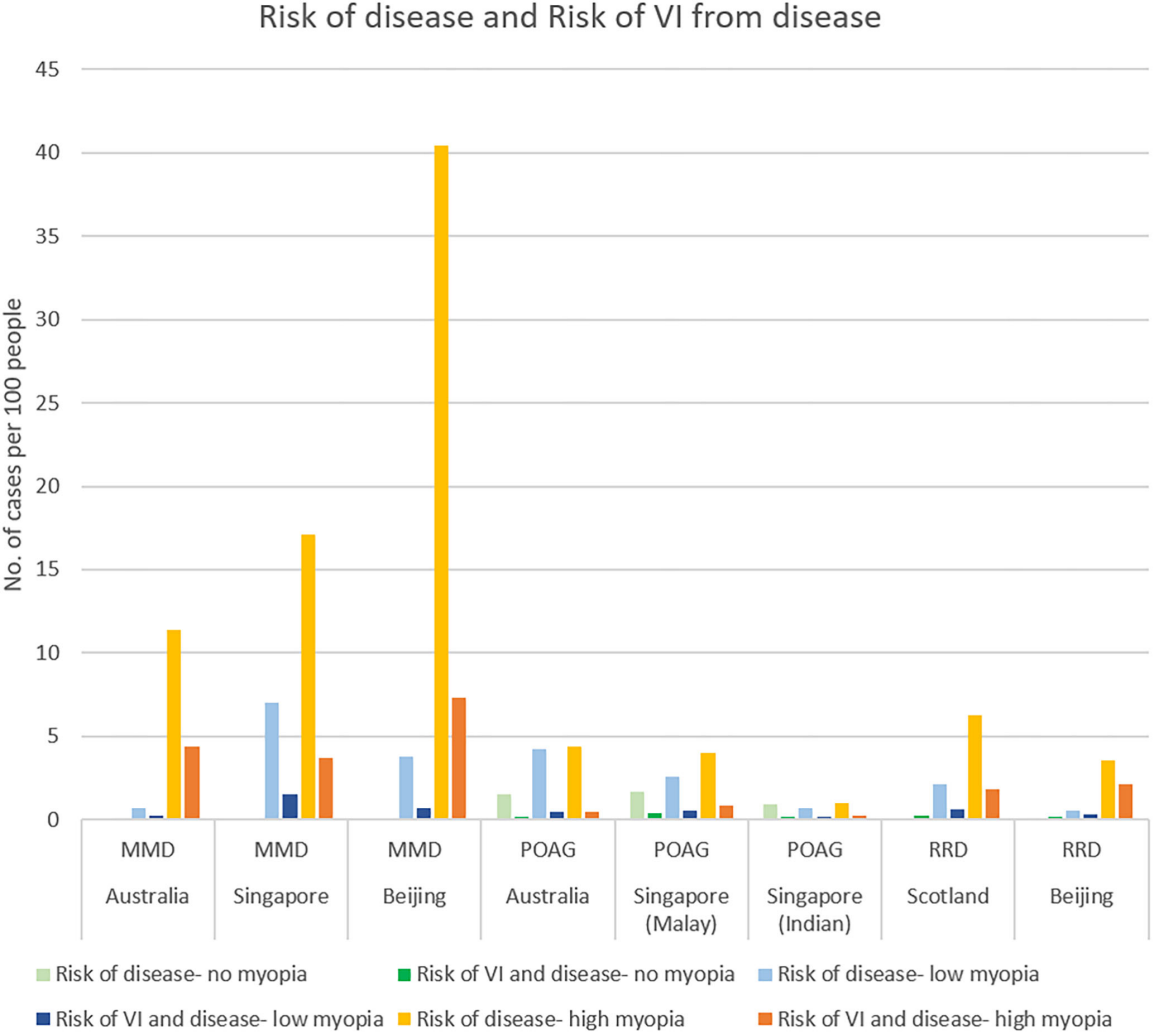
Bar graph illustrating the risks of MMD, POAG, and RRD and the risks of VI from MMD, POAG, and RRD in persons with no myopia, low myopia, or high myopia within different populations.

The risks of an individual with no myopia, low myopia, or high myopia from different populations developing MMD, POAG, RRD, and subsequent VI from these pathologies are illustrated in Figure 1. The risk of developing VI from any of these three myopia-related diseases is presented by population and myopia severity in Table 7. The risk of VI from MMD, POAG, or RRD in a non-myopic person from a predominantly White population is 0.4 in 100. For persons with low or high myopia within the same population, the risks increase to 1.4 in 100 and 6.8 in 100, respectively. A non-myopic person from a predominantly East Asian population has a 0.5 in 100 risk of developing VI from MMD, POAG, or RRD. The respective risks of persons with low and high myopia from East Asia developing VI from the aforementioned pathologies are 2.4 in 100 and 10.3 in 100.

**Table 7.** Likelihood of a Person With No Myopia, Low Myopia, or High Myopia From a Predominantly East Asian or White Population Developing VI From MMD, POAG, or RRD and Not Developing VI From MMD, POAG, or RRD

Population	Refractive Group	Proportion of People Developing VI From MMD, POAG, or RRD	Proportion of People Not Developing VI From MMD, POAG, or RRD
White	No myopia	0.4 in 100	99.6 in 100
	Low myopia	1.4 in 100	98.6 in 100
	High myopia	6.8 in 100	93.2 in 100
East Asian	No myopia	0.5 in 100	99.5 in 100
	Low myopia	2.4 in 100	97.6 in 100
	High myopia	10.3 in 100	89.7 in 100

## Discussion

Myopia is a known risk factor for ocular disease; however, the risk values reported in the literature are often presented in relative terms, which are numerically higher and therefore may exaggerate the reader's perception of the potential harm associated with the risk.^29^^,^^70^ Without the context provided by absolute values, relative risk measures can be misinterpreted. Although several studies have documented the prevalence of myopia-related diseases and the frequency of VI from the disease, this is the first time, to our knowledge, that combined measures of the risk of developing myopia-related pathologies and subsequent VI have been estimated. In accordance with NICE guidance, the data have been presented in absolute terms and using natural frequencies (e.g., 1 in 100)^30^ for a person with no myopia, low myopia, or high myopia across both predominantly White and East Asian populations.

### VI from MMD

MMD poses the greatest risk to the ocular health of individuals with high myopia, with an estimated 4.4 in 100 and 7.3 in 100 White and East Asian highly myopic persons aged over 40 years developing VI from MMD, respectively. Despite this, 95.6 in 100 White persons with high myopia and 92.7 in 100 East Asian persons with high myopia will not develop VI from MMD. The prevalence of MMD in persons with low and high myopia was higher in the studies conducted in China^28^ and Singapore^27^ compared to Australia,^7^ which could indicate that East Asian individuals have a higher genetic susceptibility to MMD. A recently published meta-analysis found no significant difference in the prevalence of MMD by location between studies conducted in Asia and non-Asian continents; however, the prevalence of MMD was significantly higher in people of East Asian ethnicity compared to European ethnicity (75.9% vs. 18.0% in those with high myopia).^71^ Notably, the pooled prevalence of MMD in East Asian persons with high myopia reported in the aforementioned study^71^ (75.9%) is almost twice as high as the prevalence of MMD reported among Chinese persons with high myopia in the Beijing Eye Study^28^ and over four times greater than the prevalence of MMD in persons with high myopia in Singapore.^27^ However, differences in the age and SER distributions among studies will influence the reported frequency of MMD. As the risk of MMD rises with increasing age and more negative SER,^6^ samples with a large proportion of older individuals or subjects with higher levels of myopia are likely to have a higher prevalence of MMD. Overall, MMD affects 1.2% of the general population over 40 years of age in Australia,^7^ and 3.1% and 3.8% of the population in Beijing^28^ and Singapore,^27^ respectively. Although this is certainly a public health concern, it is important to note that other ocular health conditions, such as age-related macular degeneration (AMD), affect a greater proportion of the population. The global prevalence of AMD in 45- to 85-year-olds is 8.69%,^72^ which is between two and seven times more prevalent than MMD.

### VI from RRD

The second largest risk of VI to individuals with high myopia was from RRD, affecting 1.9 in 100 White persons with high myopia and 2.1 in 100 East Asian persons with high myopia over the age of 40 years. The annual incidence of RRD was substantially greater in the predominantly White population than in the East Asian population. It was calculated that the annual incidences of RRD in persons with low and high myopia who are over 40 years in Scotland are 55 per 100,000 and 161 per 100,000, respectively. In contrast, the respective annual incidences of RRD in persons with low and high myopia over 40 years old in Beijing are 14 per 100,000 and 84 per 100,000. According to these data, a Scottish person with low myopia has a fourfold greater risk of RRD compared to a person with low myopia from Beijing. For an individual with high myopia, the risk of RRD is twice as likely in Scotland compared to Beijing. The reason for the variation in RRD incidence between Scotland and Beijing is not clear; however, it could be associated with the age distribution of the study populations. At the time of data collection, Scotland had almost twice the number of people over 70 years of age compared to Beijing (608,358 in Scotland^60^ vs. 376,200 in Beijing^59^). As RRD incidence increases with age and peaks at ages 60 to 70 years,^2^ a population with a larger proportion of older people will capture a greater number of RRD cases.

Interestingly, the visual outcomes of RRD are substantially poorer in East Asia compared to Scotland. In Singapore, 35.1% of RRD cases in Chinese subjects had VA of 0.5 to 1.0 logMAR, and 24.6% had a VA worse than 1.0 logMAR following surgical repair.^65^ In total, the postoperative VA of RRD patients was 0.5 logMAR or worse in almost 60% of cases.^65^ In contrast, our calculations suggest that only 29.7% of RRD cases in Scotland result in a postoperative VA of worse than 0.3 logMAR. This figure was determined based on the assumption that macula-on RRD cases are likely to retain good postoperative VA (of 0.3 logMAR or better). However, there may be a small number of macula-on RRD surgeries with poor visual outcomes; as a result, the risk of VI from RRD in White individuals may be underestimated.

### VI from POAG

Of the three diseases studied, VI from POAG carried the lowest risk to individuals with myopia, with 0.5 in 100 White persons with high myopia and 0.2 to 0.9 in 100 East Asian persons with high myopia affected. Among the Singapore Indian East Asian population, the risk of VI from POAG was found to be the same in each refractive category.

The prevalence figures extracted from the studies used in this analysis^4^^,^^47^^,^^49^ were not age standardized. However, the authors of each of the three studies provided adjusted ORs to illustrate the relationship between POAG and myopia. After adjusting for other glaucoma risk factors, the ORs and 95% confidence intervals (CIs) of a person with low and moderate–high myopia developing POAG compared to a person without myopia in the Blue Mountains Eye Study were 2.1 (CI, 1.2–3.8) and 3.3 (CI, 1.7–6.4), respectively.^4^ In the Singapore Malay Eye Study, the age- and sex-adjusted ORs and 95% CIs for developing POAG in persons with low and moderate–high myopia compared to emmetropic persons were 1.52 (CI, 0.74–3.10) and 2.91 (CI, 1.12–7.57), respectively.^47^ In the Singapore Indian Eye Study, the respective adjusted ORs and 95% CI for POAG in persons with low, moderate, and high myopia were 0.62 (CI, 0.27–1.45), 1.10 (CI, 0.23–5.36) and 6.97 (CI, 2.20–22.16).^49^ Notably, in both the Singapore Malay Eye Study^47^ and Singapore Indian Eye Study,^49^ the ORs for POAG in persons with low myopia were not statistically significant, and the wide CIs for each OR demonstrates that there is considerable uncertainty regarding the strength of the relationship between myopia severity and POAG. This could be associated with the difficulty in evaluating the presence of glaucoma in myopic eyes. The morphological features of myopic eyes, such as tilted optic discs and large disc and cup size, may lead to a number of eyes being misdiagnosed as glaucomatous.^4^^,^^73^^,^^74^ Furthermore, the IMI has questioned the nature of optic nerve damage in highly myopic eyes, suggesting that it may not be truly glaucomatous and should instead be classified as “myopia-associated glaucoma-like optic neuropathy.”^75^

### VI from MMD, POAG, or RRD

The risk of VI from MMD, POAG, or RRD was greatest in persons with high myopia, with risks of 6.8 in 100 and 10.3 in 100 in White and East Asian populations, respectively. In other words, 93.2 in 100 persons with high myopia in a White population and 89.7 in 100 persons with high myopia in an East Asian population will not develop VI as a result of MMD, POAG, or RRD. These findings are comparable to a previous study by Verhoeven et al.^76^ conducted in the Netherlands which suggests that 10.9% of individuals with high myopia (18/165; SER ≤ −6.00 D) over 55 years of age had bilateral low vision (VA > 0.5 and ≤ 1.3 logMAR) and blindness (VA > 1.3 logMAR). In a similar study of adults from the Netherlands using a larger sample, Tideman et al.^77^ reported that, by the age of 75 years, the cumulative risks of VI (VA > 0.5 logMAR) are 3% and 20% for those with low myopia (SER −0.50 and >−3.00 D) and high myopia (SER < −6.00 D to >−10.00 D), respectively. The study by Verhoeven et al. used population data from the Rotterdam Eye Study I and II, representing 9176 participants ages 55 years or older.^76^ Tideman et al.^77^ used a larger sample of 15,404 subjects 25 years or older of European descent, from the Rotterdam Studies I, II, and III; the Erasmus Rusphen Family Study; and the case–control Myopia Study. Data from both of these papers^76^^,^^77^ are commonly cited in the literature to illustrate the risk of myopia to visual function.^12^^,^^25^ However, these studies include all cases of VI in a myopic population irrespective of the cause. As a result, the analyses will include cases of VI that did not occur due to myopia-related complications and thus may be an overestimation of the direct risk of myopia to visual function. Because the present study sampled data from papers reporting the prevalence of VI in people with myopia-related pathologies, it may provide a more realistic reflection of the risk of VI associated with myopia.

**Figure 2.**
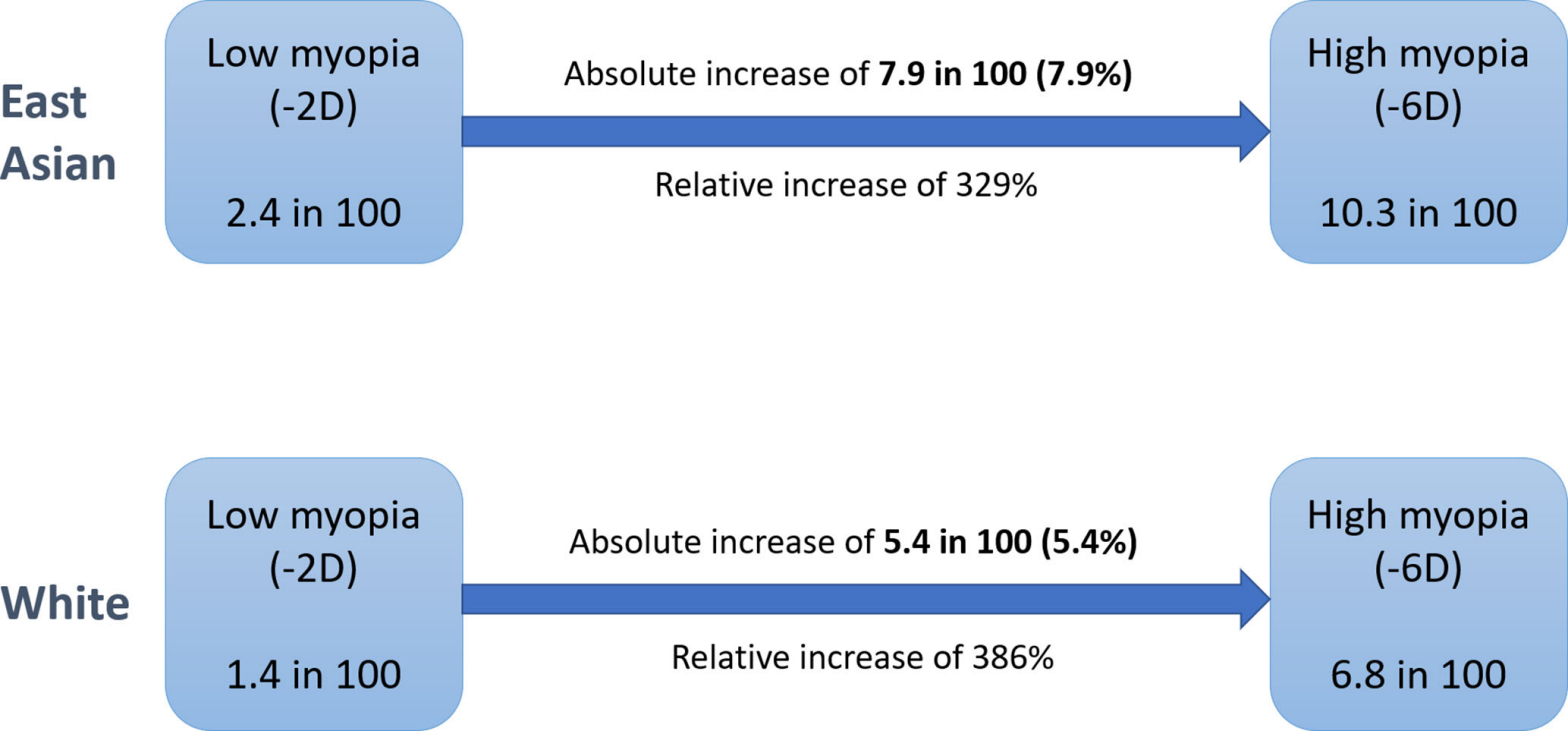
Diagram of the risk of developing VI from MMD, POAG, or RRD in persons with low or high myopia from predominantly White and East Asian populations. The difference in risk between persons with low and high myopia is expressed in both absolute and relative terms.

A paper by Bullimore et al.^25^ graphically illustrated cumulative risks of VI in a European adult population to demonstrate that the risk of VI increases with age and myopia severity. For comparison purposes, data were extracted from this graph, and the highest and lowest risks of VI (at ages 75 and 55 years, respectively) were averaged for a person with −2 D and −6 D of myopia. The absolute risks of VI for −2 D and −6 D of myopia were estimated to be an average of 1.9% and 5.5%, respectively, which are similar to the combined absolute risk of VI from MMD, POAG, or RRD calculated in the current paper for a predominantly White population (1.4% and 6.8%, for persons with −2 D and −6 D of myopia, respectively). The absolute risk difference for persons with high myopia developing VI from myopia-related diseases compared to persons with low myopia is 5.4% in the present study, compared to 3.6% using the values extracted from the paper by Bullimore et al.,^25^ who stated that every 1-D increase in myopia is associated with a 24% to 31% increase in the risk of VI.^25^ Our findings indicate a greater relative risk of myopia-related VI, of 48.8% per 1-D increase in myopia in a predominantly White population. However, although the relative risk per diopter is approximately twice as high as that reported by Bullimore et al.,^25^ the difference between the absolute risk values is much smaller. This further highlights the importance of expressing risk statistics in absolute terms. Figure 2 illustrates how the differences in risk of VI from myopia-related diseases in persons with low and high myopia compare when presented in relative and absolute terms.

### Clinical Applications

**Figure 3.**
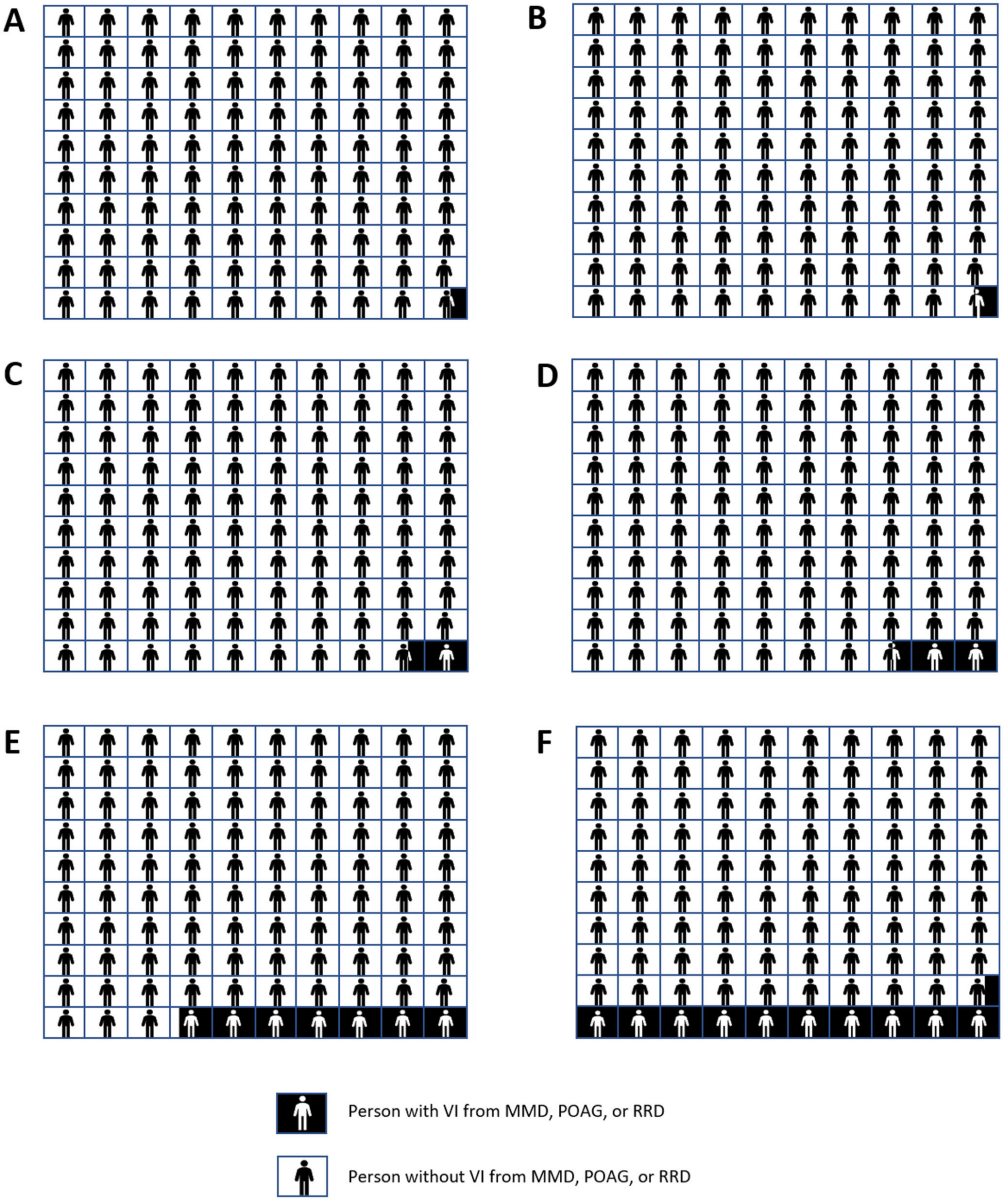
Decision tool in the form of pictographs illustrating the absolute risks of a person without myopia, with low myopia (−2.00 D), or high myopia (−6.00 D) in East Asian and White populations developing VI from MMD, POAG, or RRD. Grids **A**, **C**, and **E** represent a White population with no myopia, low myopia, or high myopia, respectively. Grids **B**, **D**, and **F** represent an East Asian population with no myopia, low myopia, or high myopia, respectively.

In clinical practice, effective risk communication is vital to allow patients to make an informed decision about their health care.^30^ The interpretation of risk is complex and can be influenced by the use of descriptive terms, framing effects, and individual experience.^70^ Practitioners should be mindful of this when communicating the risks of myopia complications to patients and consider a tailored approach, which could involve the use of visual decision aids such as bar graphs or pictographs (Figure 3).^30^^,^^70^ As well as explaining the risk of developing VI from myopia-related diseases, the probability of *not* developing VI should also be outlined^30^ to help patients put the risk into context.

Reducing the risk of complications in adulthood is a key motivating factor for prescribing myopia management interventions in childhood^78^; however, practitioners should consider the expected cumulative efficacy of treatments alongside the difference in absolute risk, not just relative risk, that can be achieved by reducing myopia severity. The probability of a child developing high myopia could be incorporated into the risk calculation using data from axial length percentile growth curves.^79^ For example, if a White male child aged 11 years old in the 98th percentile has a 31% risk of developing high myopia^79^ and the risk of VI from MMD, POAG, or RRD in a person with high myopia is 6.8 in 100, then the child's overall risk of developing high myopia and future VI from any myopia-related disease is 2.1 in 100. As well as a potential reduction in disease risk, a number of other benefits associated with slowing the progression of myopia should be considered, such as improved spectacle cosmesis, reduced dependence on spectacles, and a general increase in quality of life.^80^

### Considerations

There are some important considerations that need to be borne in mind when interpreting the absolute risk values reported in this study. First, the risk estimations for East Asian and White populations were calculated using data extrapolated from individual studies and therefore are not a full representation of each population group. For some risk calculations, it was not possible to determine disease and VI prevalence data from the same population; in these instances, an alternative population with similar demographics was sourced. A larger meta-analysis of the prevalence of myopia-related diseases and VI could be conducted to pool risk data from multiple regions and ethnic groups; however, pooling data could lead to errors associated with the heterogeneity of studies. Notably, 95% CIs were not provided for the risk estimates, as it was not possible to calculate these from the data provided from the studies included in the analysis. A further limitation of the study is that many of the prevalence figures used to establish myopia-related disease risk were not age standardized; therefore, differences in the age distribution between people with low and high myopia may have influenced the results. Similarly, variation in the refractive distribution of populations and the refractive error classifications used by different studies may have influenced the reported prevalence of myopia-related diseases and consequently, the risk estimates produced in this study.

It was noted that there were methodological differences between studies reporting the prevalence of VI and disease, such as the use of different disease definitions and classifications of VI, which may have led to variation in the reported prevalence figures. In our analysis of MMD risk, two studies^7^^,^^28^ used the same criteria for identifying cases of MMD, whereas the third study^27^ used the Meta-Analysis for Pathological Myopia (META-PM) classification. The latter has a less stringent definition of chorioretinal atrophy and may have led to over-reporting of MMD cases; however, studies testing this hypothesis show conflicting results.^6^^,^^71^ The majority of the studies included in the risk calculation adhered to the VA threshold for mild VI outlined in the ICD-11 guidelines.^32^ However, although VI is typically classified based on the VA of the better seeing eye,^81^ some authors chose to categorize VI based on the proportion of eyes that met the VA criteria or the proportion of subjects who met the VA threshold in at least one eye. In both of these instances, the reported rate of VI is likely to be greater than if using the presenting VA in the better eye. Therefore, the reported risk of VI from myopia-related diseases may be overestimated in some instances.

Although there is no clearly defined age threshold at which myopia-related complications occur, a recent paper from the IMI suggests that the prevalence and severity of pathological myopia increase substantially from the age of 40 years onward.^82^ It is also known that the likelihood of developing diseases such as MMD, POAG, and RRD increases with increasing age.^83^ Indeed, in the Singapore Epidemiology of Eye Diseases study, the prevalence of MMD amongst persons with high myopia ranged from 25.7% in those under 70 years old to 65% in those 70 years and older.^27^ Similar findings were reported in a recent study conducted in Russia, which found an MMD prevalence of 75% among highly myopic persons over 85 years old.^84^ In contrast, the prevalence figures and subsequent risk estimates reported within this study are associated with populations 40 years of age and over, representing a broad time frame during which individuals with myopia are likely to experience myopia-related diseases and VI. As these figures represent the risk of developing VI from a myopia-related disease during several years of adulthood (i.e., from the age of 40 years onward), the estimates are likely to underestimate the risk for people at the higher end of the age range and overestimate the risk for people at the lower end of the age range. An alternative method of evaluating risk that encompasses the inherent age-related differences in disease prevalence over time is to calculate the cumulative lifetime risk of myopia-related disease and subsequent VI. Unfortunately, it was not possible to comprehensively evaluate the cumulative lifetime risk of VI from MMD, POAG, or RRD within different populations in the present study due to a lack of published data on the age-disease profiles within different refractive categories. Only two of the studies identified in the literature review provided data on the prevalence of the disease by age within each refractive group, and none provided data on the proportion of those with the disease with VI categorized by age. Nonetheless we suggest that the risk of VI across the period of adulthood at which complications are likely to occur is the outcome of interest when considering a child's future risk of VI from a myopia management perspective.

Interestingly, although myopia is associated with an increased risk of VI from MMD, POAG, and RRD, there is evidence to suggest that persons with myopia have a reduced risk of VI from other conditions. For example, children with myopia are less likely to have amblyopia than those with hyperopia,^85^^,^^86^ and adults with myopia are less likely to develop angle-closure glaucoma.^87^ Furthermore, data from the Singapore Indian Eye Study suggest that the prevalence of AMD is lower in persons with myopia compared to other refractive groups.^49^ In this study, 1.6% of participants with myopia were diagnosed with AMD, compared to 3.3% and 4% of subjects with emmetropia and hyperopia, respectively.^49^ Similar findings have been reported in a recent paper by Jonas et al.^87^ It is unclear, however, whether the reduced risk of these conditions in persons with myopia outweighs the increased risk for diseases such as MMD, POAG, and RRD.

## Conclusions

Absolute risk data are necessary to provide context to relative risks and to avoid perceived over-exaggeration of risks. The absolute risks of persons with no myopia, low myopia, or high myopia developing VI from MMD, POAG, or RRD have been presented here for the first time, to our knowledge, along with visual decision aids to facilitate risk communication in practice.

Three key findings include the following:

1. Visual impairment from myopic macular degeneration is the greatest risk to the ocular health of persons with high myopia, affecting 4.4 in 100 White persons with high myopia and up to 7.3 in 100 East Asian persons with high myopia.
2. The absolute risks of developing visual impairment from any myopia-related disease (MMD, POAG, or RRD) are 0.4 in 100 for a person with no myopia, 1.4 per 100 for a person with low myopia, and 6.8 in 100 for a person with high myopia in a predominantly White population. The same risks in an East Asian population are 0.5 in 100, 2.4 in 100, and 10.3 in 100 for an individual with no myopia, low myopia, or high myopia, respectively.
3. Absolute values can aid our understanding of disease risk by providing context to relative risks. Consequently, absolute values should be used when communicating the risk of myopia-related diseases to patients. Decision tools, such as pictographs, can help facilitate risk communication.
